# A Tiered Genetic Screening Strategy for the Molecular Diagnosis of Intellectual Disability in Chinese Patients

**DOI:** 10.3389/fgene.2021.669217

**Published:** 2021-09-23

**Authors:** Limeng Dai, Danyan Zhang, Zhifeng Wu, Xingying Guan, Mingfu Ma, Lianbing Li, Yuping Zhang, Yun Bai, Hong Guo

**Affiliations:** ^1^ Department of Medical Genetics, College of Basic Medical Science, Army Medical University, Chongqing, China; ^2^ Chongqing Population and Family Planning Science and Technology Research Institute/NHC Key Laboratory of Birth Defects and Reproductive Health, Chongqing, China; ^3^ Department of Pediatrics, Xinqiao Hospital, Army Medical University, Chongqing, China

**Keywords:** whole exome sequencing (WES), chromosomal microarray (CMA), copy number variation (CNV), developmental delays (DD), intellectual disability (ID)

## Abstract

**Objective:** Intellectual disability (ID) is one of the most common developmental disabilities. To identify the genetic etiology of IDs in Chongqing, we conducted a multistage study in Chinese Han patients.

**Methods:** We collected the clinical and etiological data of 1665 ID patients, including 1,604 from the disabled children evaluation center and 61 from the pediatric rehabilitation unit. Routine genetic screening results were obtained, including karyotype and candidate gene analysis. Then 105 idiopathic cases with syndromic and severe ID/developmental delay (DD) were selected and tested by chromosomal microarray (CMA) and whole exome sequencing (WES) sequentially. The pathogenicity of the CNVs and SNVs were evaluated according to ACMG guidelines.

**Results:** Molecular diagnosis was made by routine genetic screening in 216 patients, including 196 chromosomal syndromes. Among the 105 idiopathic patients, 49 patients with pathogenic/likely pathogenic CNVs and 21 patients with VUS were identified by CMA. Twenty-six pathogenic CNVs underlying well-known syndromic cases, such as Williams-Beuren syndrome, were confirmed by multiplex ligation-dependent probe amplification (MLPA). Nine novel mutations were identified by WES in thirty-fix CNV-negative ID cases.

**Conclusions:** The study illustrated the genetic aberrations distribution of a large ID cohort in Chongqing. Compared with conventional or single methods, a tiered high-throughput diagnostic strategy was developed to greatly improve the diagnostic yields and extend the variation spectrum for idiopathic syndromic ID cases.

## Introduction

Intellectual disability (ID) or developmental delay (DD) is one of the most common reasons for visiting pediatric rehabilitation or genetic counseling clinics. The incidence is estimated at over 1% worldwide, which seriously endangers the physical and mental health of children (Moeschler and Shevell, 2014). ID is characterized by intellectual and adaptive deficits, such as poor understanding of language, low learning ability, and limited social and practical activities. In addition to a few straight-forward ID cases, ID is often accompanied by other clinical symptoms or systematic malformations, which seriously affects quality of life in this population. Genetic abnormalities are one of the most important causes of IDs (Ropers, 2008; Moeschler and Shevell, 2014; Vissers et al., 2016). History investigation, clinical phenotype analysis, and conventional auxiliary laboratory techniques, including karyotype analysis and candidate gene screening, confirm the diagnosis of IDs. However, there is still a large number of patients without an etiologic diagnosis. This creates a heavy burden in medical costs and social stress on the families. Chongqing is in the southwest of China, with distinctive regional, ethnic, and economic characteristics. ID is one of the most important etiological components of birth defects in this district. It is very important to illustrate the epidemiological characteristics and genetic etiology of ID in this district.

The genetic etiology of ID is not well described. Traditional karyotype analysis, metabolic analysis, and candidate gene screening can only solve about 30% of the causes of genetic IDs in clinical practice (Ropers, 2008). Except chromosomal abnormalities or well-known gene mutations, previous studies of ID patients only confirmed the causative role for small copy number variations (CNVs) in the pathogenesis (Merikangas et al., 2009; Miller et al., 2010; Mefford et al., 2012). Due to its main advantages, chromosomal microarray (CMA) has facilitated the discovery of novel rare DNA CNVs across the genome. So, CMA testing has been recommended as a first-tier cytogenetic diagnostic test for patients with ID or multiple congenital anomalies (Miller et al., 2010). However, the interpretation of CNVs is very challenging (Merikangas et al., 2009). The detection of balanced rearrangements and single nucleotide variations (SNVs) are also beyond the capability of CMA analysis. In recent years, next generation sequencing (NGS) has enabled identification of multiple genetic variations, which play important roles in the pathogenesis of IDs (Harripaul et al., 2017; Bruel et al., 2020). So, the application of high-throughput technologies, including CMA and whole exome sequencing (WES), has become an effective strategy for genetic analysis in IDs (Fell and Nagy, 2021).

In the study, we recruited 1604 ID patients from the disabled children evaluation center. After review of the routine genetic screening results, 216 cases obtained a genetic diagnosis. For the remaining 44 undiagnosed syndromic ID cases and other 61 idiopathic severe ID/DD cases from the pediatric rehabilitation unit, we conducted a sequential approach by using CMA and WES. The results revealed that this might be a general strategy for the molecular screening of IDs. With the idiopathic ID cases, clinical application of high-throughput techniques greatly improved the diagnostic yields. Several novel mutations or genomic regions of clinical significance were also identified, which enriched the genotype-phenotype correlations in the ID patients and provided clues for the exploration of neurodevelopmental genes.

## Materials and Methods

### Patients

This study was performed with the approval of the Ethics Committee of Army Medical University, Chongqing, China. A written statement of informed consent was obtained from the legal guardian of each patient in the study. The subjects were treated in accordance with the tenets of the Declaration of Helsinki. Undergoing a diagnostic evaluation of ID, 1,604 cases were collected from the disabled children evaluation center between January 2013 and December 2016. The inclusion criteria contained the following characteristics: 1) learning disability, 2) language barriers, 3) autistic features or suitability barriers, 4) may have other developmental delays, such as growth or motor delays, and 5) may have congenital multiple malformations. The other 61 participants were identified and enrolled in the pediatric rehabilitation unit at the Xinqiao Hospital of Army Medical University from June 2013 to December 2020. The medical records of all the patients were reviewed retrospectively. Tabulated data of each patient included: 1) demographic information: age, gender, family history, history of birth, growth and development history, and systemic disorders; 2) details of the ID features: presence of malformations, intelligence quotient scoring (IQs); 3) other neuropsychiatric phenotypes (i.e., epilepsy, attention deficit and hyperactivity disorder, autism spectrum disorders, and schizophrenia).

### Routine Genetic Screening

#### Karyotyping

Cells were incubated at 37°C in MEM (Gibco/Life Technologies, USA) containing phytohemagglutinin for 72 h, then colcemid (0.2 μg/ml) was added for a further 40 min of incubation. The dividing cells were processed in 0.075 M of KCl and fixed in 3:1 methanol–acetic acid. Giemsa banding was used to produce a visible 550-band resolution karyotype on the slides. The chromosomes were analyzed and reported by experienced cytogeneticists manually, according to the recommendations of the International System for Human Cytogenomic Nomenclature.

#### Candidate Gene Analysis

Triplet primed PCR (TP-PCR) was performed to screen mutations in the FMR1 gene according to Chen’s protocol (Chen et al., 2010). The concentrations of 11 amino acids, 31 acylcarnitines, and 1 ketone succinylacetone were measured by tandem mass spectrometry. Individuals with clear aberrant initial screening results were referred to confirmatory tests, including biochemical and genetic analysis.

### DNA Extraction and Sanger Sequencing

DNA was extracted from peripheral blood leukocytes of the patients by using the Wizard Genomic DNA Purification Kit (Promega, US). When possible, parental DNA was collected. The quantity and quality of DNA were determined by using NANODROP 1000 (Thermo Fisher, US). Variants identified by the exome sequencing were confirmed by Sanger DNA sequencing. The software Primer3 was used to design the primers. PCR conditions and the primer pairs are available upon request. DNA sequences were analyzed using the vector NTI 11.0 software package. The DNA mutation numbering system we used is based on a cDNA sequence with +1 corresponding to the A of the ATG translation initiation codon in the reference sequence.

### CMA Platform and MLPA Assay

The probands were screened *via* CMA to detect the genome-wide CNVs. The CMA assay was conducted by the KingMed Diagnostics Corporation (Guangzhou, China), by using the Affymetrix CytoScan HD array (Thermo Fisher Scientific, US), according to the manufacturer’s instructions. Commercial reference DNA (male and female) provided by Thermo Fisher Scientific were used for the analysis. Genotype and CNV identification and an assessment of genotyping integrity were conducted by using Affymetrix Chromosome Analysis Suite software version 3.1 (Thermo Fisher Scientific, US).

The genomic regions are described according to the GRCh37/hg19 reference sequence (University of California Santa Cruz). The significance of each CNV was determined by comparison to the public database, such as the Database of Genomic Variant (DGV http://dgv.tcag.ca/dgv/app/home) and DECIPHER database (http://decipher.sanger.ac.uk/). When available, blood samples were obtained from patient’s parents and the same analysis was done to investigate the inheritance of CNVs. Microdeletions and microduplications were evaluated according to American College of Medical Genetics (ACMG) guidelines (Riggs et al., 2020).

For confirmation of some pathogenic CNVs, we selected the commercial MLPA probes-targeted regions associated with 23 well-known microdeletion or microduplication syndromes (including Williams-Beuren syndrome, Prader-Willi/Angelman syndrome, DiGeorge syndrome, Xq28 duplication syndrome, and Rett syndrome, etc). According to the manufacturer’s instructions, MLPA was performed on the proband’s genome by using the SALSA MS-MLPA kit P245-B1 (MRC-Holland, Netherlands).

### 2.5. Whole Exome Sequencing

The genomic DNA of the proband was fragmented to generate 200–300 bp insert fragments. The paired-end libraries were prepared following the Illumina library preparation protocol. The exome was captured using the SureSelect Human All Exons Plus kit (Agilent, Santa Clara, US). Paired-end sequencing was carried out on an Illumina HiSeq 3,000 sequencer (Illumina, San Diego, US).

Raw image files were processed by the Illumina Pipeline for base calling using default parameters. Primary data came in fastq form after image analysis and base calling was conducted using the Illumina Pipeline. The data were filtered to generate “clean reads” by removing adapters and low quality reads. The raw results were analyzed by using a customized pipeline that utilized published algorithms in a sequential manner. Sequencing reads were mapped to the reference human genome version hg19 (http://genome.ucsc.edu/). Variant analysis was performed using SOAPsnp software and Samtools for SNPs and indels, respectively. All SNPs were identified by using the dbSNP, HapMap, 1,000 human genome dataset (http://www.1000genomes.org/), and a local database developed by BGI (Shenzhen, China).

### Bioinformatics Analysis of the SNVs and CNVs

Several online prediction software programs were used to predict the functional significance of the SNVs, including PolyPhen 2 (http://genetics.bwh.harvard.edu/pph2), SIFT (http://sift.jcvi.org), MutationTaster (http://www.mutationtaster.org/), and I-Mutant v2.0 (http://folding.biofold.org/i-mutant/i-mutant2.0.html). The pathogenicity of the SNVs was evaluated according to American College of Medical Genetics guidelines (ACMG) (Richards et al., 2015).

For CNVs of uncertain clinical significance (VUS), the genes in the region were analyzed by their brain expression profiles in GTEx (https://www.gtexportal.org) and HBT (https://hbatlas.org/), their GO biological processes in SYNGO (https://www.syngoportal.org) and HPO terms (https://hpo.jax.org/app), and their contributions to mouse phenotypes in MGI (https://phenome.jax.org/).

## Results

### Clinical Information of the Cohort

In total 1,665 unrelated ID patients were included; the male/female ratio was 2.86:1. IQ scoring was carried out by using standard test scales for children (WISC III and WISC IV) (Baron, 2005). The severity was assessed also referring to the criteria of Diagnostic and Statistical Manual of Mental Disorders, 5th Edition (DSM-5). IQ scores of the patients revealed mild (23.59%), moderate (43.15%), and severe (33.26%) ID. Some of the patients also exhibited general or facial dysmorphism (39.68%), speech delay (46.74%), psychomotor retardation (39.16%), social dysfunction (23.41%), or growth retardation (14.62%). Among these patients, three had a family history of ID, while the others had no obvious family history.

**FIGURE 1:**
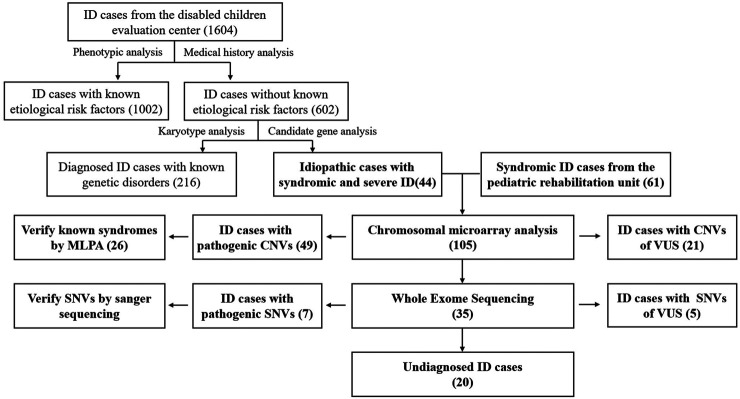
Algorithmic approach for patients with intellectual disability (ID).

For the 1604 ID patients from the disabled children evaluation center, 1,002 cases (62.47%) were found to experience known etiological risk factors, such as birth injury or infection. The routine genetic screening results from 602 cases revealed that 216 cases (35.88%) obtained a genetic diagnosis. Of which, 196 patients had chromosome number or structural abnormality. Down syndrome (27.57%, 166/602) and Turner syndrome (2.99%, 18/602) were the most common diseases. Twenty patients were diagnosed with monogenic disorders mainly composed of Fragile X syndrome (1.66%, 10/602) and inherited metabolic diseases. According to the inclusion criteria a-e in ID patients, 44 undiagnosed syndromic severe ID cases were selected from the remaining undiagnosed cases. The other 61 idiopathic severe ID/DD cases were also recruited from the pediatric rehabilitation unit. The blood samples of the 105 cases were obtained and examined according to a multi-step genetic diagnostic procedure, including CMA and WES sequentially (Figure 1).

### Detection Rate of Genetic Defects

Among the 105 idiopathic ID patients, pathogenic/likely pathogenic CNVs for ID were identified in 46.67% of patients (49/105), according to the international guidelines of ACMG (Riggs et al., 2020). The variations of uncertain clinical significance (VUS) were detected in 20% of cases (21/105) and benign CNVs in 33.33% of cases (35/105). Validation testing was performed in the 26 patients with pathogenic CNVs (53.06%, 26/49) by MLPA. In the 35 patients with benign CNVs, 16 mutations were identified in 12 patients (11.43%, 12/105) by using WES, including 11 pathogenic or likely pathogenic mutations associated with known monogenic disorders. Nine novel SNVs were reported for the first time in ID cases. For the 105 idiopathic ID cases, 56 were further diagnosed by using high-throughput techniques.

### Classification and Characteristics of CNVs

As shown in Table 1, a total of 49 patients were found to carry pathogenic or likely pathogenic CNVs, which included 1 UPD, 39 deletions, and 17 duplications, ranging from 21 Kb to 35.3 Mb. Each patient had 1.16 pathogenic CNVs on average. Microdeletions were more frequent than microduplications (68.42 vs. 29.82%), however, the average size did not differ significantly. Forty patients (81.63%) were identified with only one clinically relevant CNV. For the recurrent microdeletion or microduplication syndromes, the most common pathogenic CNVs were 15q11.2-q13.1 deletion (#105830 or #176270, 5 cases), 7q11.23 deletion (#194050, 4 cases), MECP2 duplication/deletion (#300260 or #312750, 4 cases), 16p11.2 deletion (#611913 or #613444, 3 cases), 22q11.21 deletion (#188400, 2 cases), 5p^−^ (#123450, 2 cases), 1p36 deletion syndrome (#607872, 2 cases), and 1q43-q44 deletion syndrome (#612337, 2 cases). Eight patients (8/49, 16.33%) had two pathogenic CNVs. Seven of them had one deletion and one duplication. There was only one patient with two deletions. Most of the pathogenic CNVs (49/57, 85.96%) were larger than 500 kb, whereas eight pathogenic CNVs smaller than 500 kb were identified. They included three patients with MECP2 duplications.

**TABLE 1:** Pathogenic and likely pathogenic genomic CNVs identified by CMA in 49 patients with ID.

Case	Age	Sex	CMA results (ISCN (2013)	Imbalance	Inheritance	Size	Known syndrome
#51	6 Y	M	arr[hg19]1p36.32-p36.33(849,466–2,579,267)×1	deletion	Unknown	1.7 Mb	1p36 deletion syndrome
#14	6 M	F	arr[hg19]1p35.3–36.11(27,476,565–29,579,157)×1	deletion	Unknown	2.1 Mb	1p36 deletion syndrome
#13	21 M	F	arr[hg19]1q21.1-1q21.2(146,105,170–147,830,830)×1	Deletion	Unknown	1.7 Mb	1q21.1 deletion syndrome
			arr[hg19]1q43-qter(243,653,445–249,206,548)×1	deletion		5.6 Mb	/1q43-q44 deletion syndrome
#26	14 M	F	arr[hg19]1q42.3-q43(235,251,057–242,239,118)×1	deletion	Unknown	7.0 Mb	1q43-q44 deletion syndrome
#96	9 M	F	arr[hg19]2p25.1-pter(12,770–11,050,573)×3	Duplication	Unknown	15.4 Mb	2p25.3 duplication syndrome/9p deletion syndrome
			arr[hg19]9p23-pter(236,119–15,714,811)×1	deletion		11 Mb	
#89	9 Y	F	arr[hg19]2q23.1(148,762,754–148,849,973)×1	deletion	De novo	87.22 Kb	2q23.1 deletion syndrome
#28	6 Y	F	arr[hg19]2q24.3-q31.1 (167,792,076–174,941,001)×1	deletion	Unknown	7.1 Mb	Unknown Dimitrov et al. (2011)
#41	12 M	F	arr[hg19]2q37.2-q37.3(236,925,786–242,783,384)×1	deletion	Unknown	5.9 Mb	2q37 deletion syndrome
#20	5 Y	F	arr[hg19]3p26.1-p26.3(61,891–6,037,368)×1	Deletion	De novo	5.9 Mb	3pter-p25 deletion syndrome (3p-)/
			arr[hg19]15q25.2-q26.3(82,231,042–102,429,112)×3	duplication		20 Mb	Unknown Roggenbuck et al. (2004)
#9	11 M	M	arr[hg19]4p16.3 (68,345–3,891,984)×1	Deletion duplication	De novo	3.82 Mb	Wolf-Hirschhorn Syndrome (4p-)
			arr[hg19]11p15.4-p15.5(230,615–3,410,609)×3			3.18 Mb	/Beckwith-Wiedemann syndrome
#40	neo	F	arr[hg19]5p13.2-p15.33(113,576–34,658,965)×1	deletion	Unknown	34.5 Mb	Cri-du-chat syndrome (5p-)
#104	5 Y	F	arr[hg19]5p15.33-pter(113,576–2,361,972)×1	Deletion	De novo	2.25 Mb	Cri-du-chat syndrome (5p-)
			arr[hg19]16q23.2-qter(79,281,580–90,155,062)×3	duplication		10.85 Mb	/Unknown Hellani et al. (2010)
#18	7 Y	F	arr[hg19]5q14.3(88,196,115–90,485,685)×1	deletion	Unknown	2.3 Mb	5q14.3 deletion syndrome
#54	20 M	M	arr[hg19]5q35.3(178,587,978–180,719,789)×1	Deletion	De novo	2.1 Mb	Unknown Rauch et al. (2003)
			arr[hg19]9q34.3(137,753,339–141,020,389)x3	duplication		3.3 Mb	/Unknown Bonati et al. (2019)
#61	24 M	F	arr[hg19]7q11.23(72,700,524–74,142,190)×1	deletion	Unknown	1.4 Mb	Williams-Beuren syndrome
#65	13 M	F	arr[hg19]7q11.23(72,701,018–74,142,190) ×1	deletion	Unknown	1.4 Mb	Williams-Beuren syndrome
#76	36 M	M	arr[hg19]7q11.23(72,718,277–74,141,603) x1	deletion	Unknown	1.4 Mb	Williams-Beuren syndrome
#70	6 Y	M	arr[hg19]7q11.23(72,718,277–74,142,190) x1	deletion	Unknown	1.4 Mb	Williams-Beuren syndrome
#73	36 M	M	arr[hg19]7q11.3(72,725,760–74,146,858) ×3	duplication	Unknown	1.4 Mb	7q11.23 duplication syndrome
#68	5 Y	M	arr[hg19]7q21.13-q31.1(88,264,345–112,848,629)×3	duplication	De novo	24 Mb	Unknown Mégarbané et al. (2000)
#103	19 M	M	arr[hg19]7q34-q36.3(141,687,233–159,119,707)×1	deletion	De novo	17.4 Mb	Unknown Rush et al. (2013)
#47	8 M	M	arr[hg19]8p11.22-p23.1(12,528,482–38,777,146)×3	Duplication	De novo	26.2 Mb	Kabuki syndrome
			arr[hg19]8p23.1-p23.3(158,048–6,999,114)×1	deletion		6.8 Mb	/Unknown Akcakaya et al. (2017)
#85	16 M	F	arr[hg19]9p22.3-pter(203,861–14,270,651)×1	deletion	Unknown	14.07 Mb	9p deletion syndrome
#27	36 M	M	arr[hg19]9q34.3(139,937,684–140,652,571)x1	deletion	Unknown	715 Kb	Kleefstra syndrome
#11	11 M	F	arr[hg19]10p14-p15.3(100,026–6,710,183)x1	Deletion	De novo	6.6 Mb	Unknown Melis et al. (2012)
			arr[hg19]18p11.31-p11.32(136,226–6,406,733)x3	duplication		6.3 Mb	/Unknown Balasubramanian et al. (2016)
#56	12 M	F	arr[hg19]10q24.2-qter(100,138,329–135,427,143)×3	duplication	De novo	35.3 Mb	Unknown Holder-Espinasse et al. (2019)
#97	7 Y	F	arr[hg19]11p15.5(1,821,840–1,977,019)x1	deletion	Unknown	155 Kb	Beckwith-Wiedemann syndrome
#77	4 M	F	arr[hg19]14q13.1-q21.1(33,448,540–39,698,995)x1	deletion	De novo	6.25 Mb	14q11-q22 deletion syndrome
#35	48 M	F	arr[hg19]15q11.2-q26.3(22,752,398–102,429,049)hmz	UPD	De novo	79.7 Mb	Angelman syndrome
#31	32 M	M	arr[hg19]15q11.2-q13.1(22,770,421–28,823,722)x1	deletion	Unknown	6.1 Mb	Angelman syndrome
#8	5 Y	M	arr[hg19]15q11.2-q13.1(22,770,421–29,057,676) x1	deletion	Unknown	6.3 Mb	Angelman syndrome
#49	11 M	M	arr[hg19]15q11.2-q13.1(23,620,191–28,522,838)×1	deletion	De novo	4.9 Mb	Prader-Willi syndrome
#102	9 M	M	arr[hg19]15q11.2-q13.1(22,770,421–30,386,399)×1	deletion	Unknown	7.6 Mb	Prader-Willi syndrome
#16	12 M	M	arr[hg19]15q11.2-q13.1(22,770,421–29,069,001)×1	deletion	Unknown	6.3 Mb	Prader-Willi syndrome
#5	5 Y	F	arr[hg19]15q11.1–13.1(20,102,541–28,525,460)×3	duplication	Unknown	8.42 Mb	15q11-q13 duplication syndrome
#33	6 M	M	arr [hg19]15q13.3 (30,386,398–32,444,044)×3	duplication	De novo	2.06 Mb	Unknown Zhou et al. (2016)
#52	5 Y	M	arr[hg19]15q25.2-q26.3(82,509,824–102,429,112)x3	duplication	De novo	21.9 Mb	Unknown Leffler et al. (2016)
#84	8 Y	F	arr[hg19]16p11.2(28,819,028–29,051,191)×1	deletion	Unknown	232 Kb	16p11.2 deletion syndrome
#59	5 Y	F	arr[hg19]16p11.2(29,571,954–30,202,125)×1	deletion	De novo	630.17 Kb	16p11.2 deletion syndrome
#66	36 M	M	arr[hg19]16p11.2(29,567,295–30,144,883)×1	deletion	Unknown	578 Kb	16p11.2 deletion syndrome
#24	5 Y	M	arr[hg19]16p13.11(14,901,699–16,473,479)×3	Duplication	Pat	1.57 Mb	Unknown Allach El Khattabi et al. (2020)
#101	5 Y	M	arr[hg19]17q21.31(44,188,378–44,254,379)×1	deletion	Unknown	66 Kb	Koolen de Vries syndrome
#21	6 M	F	arr[hg19]19q13.32(46,064,695–48,178,154)×1	deletion	De novo	2.1 Mb	Unknown Castillo et al. (2014)
#91	26 Y	M	arr[hg19]22q11.21(18,916,842–21,800,797)×1	deletion	Unknown	2.88 Mb	DiGeorge syndrome
#37	18 M	M	arr[hg19]22q11.21(19,000,000–21,401,539)×1	deletion	Unknown	2.4 Mb	DiGeorge syndrome
#3	48 M	M	arr[hg19]Xq28(153,005,690–153,438,781)x2	duplication	Unknown	433 Kb	MECP2 duplication syndrome
#44	12 M	M	arr[hg19]Xq28(153,171,615–153,622,204)x2	duplication	De novo	451 Kb	MECP2 duplication syndrome
#82	19 M	M	arr[hg19]Xq28(152,916,853–153,408,930)x2	duplication	De novo	492 Kb	MECP2 duplication syndrome
#88	36 M	F	arr[hg19]Xq28(153,282,927–153,303,631)x1	deletion	De novo	21 Kb	Rett syndrome

Age: neo, neonate, M, months, Y, years; Sex: M, male, F, female; Inheritance: Mat, inherited from the mother; Pat, inherited from the father.

In 14 patients, we identified rarely reported likely pathogenic CNVs, which were not associated with any of the known syndromes. They included regions that did not completely overlap with those of known genomic imbalances. Although all the 17 likely pathogenic (LP) CNVs were not found in healthy individuals or in DGV (http://dgv.tcag.ca/dgv/app/home). They have been identified in more than one ID patients before (Mégarbané et al., 2000; Rauch et al., 2003; Roggenbuck et al., 2004; Hellani et al., 2010; Dimitrov et al., 2011; Melis et al., 2012; Rush et al., 2013; Castillo et al., 2014; Balasubramanian et al., 2016; Leffler et al., 2016; Zhou et al., 2016; Akcakaya et al., 2017; Bonati et al., 2019; Holder-Espinasse et al., 2019; Allach El Khattabi et al., 2020). To determine the inheritance pattern, the parental DNA was available from the parent in 21 cases. We identified 20 *de novo* aberrations. The 1.57 Mb duplication identified in case #24 with was inherited from his healthy father. The other CNVs were of unknown inheritance, because of the absence of parental DNA samples.

**FIGURE 2:**
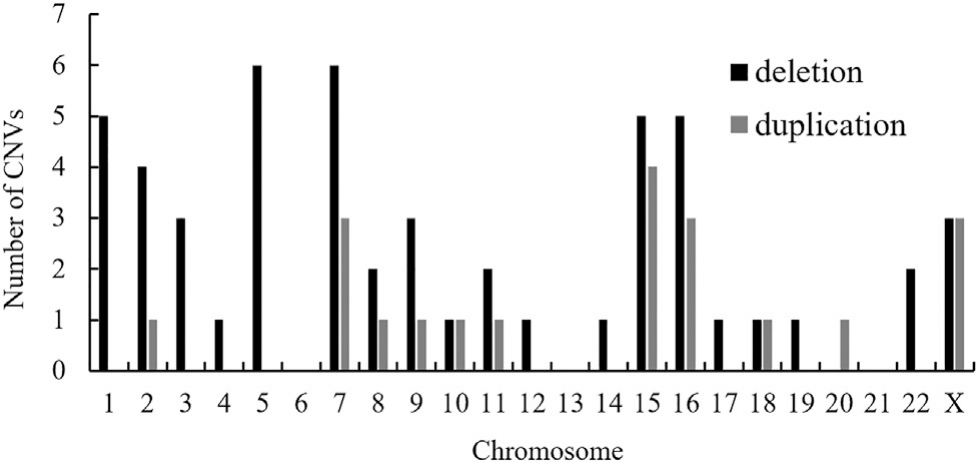
Chromosomal distribution of CNVs identified in the present study. Abbreviation: CNV, copy number variation.

Also, a total of 17 structural CNVs were classified as VUS according to the ACMG guideline, which were identified in 16 patients. Most of the VUS (12/22, 54.55%) were smaller than 500 kb, except five UPDs over 5 Mb. Among 16 cases of VUS, the origins have not been assessed. In detail there were 14 deletions (58.82%), three duplications (11.77%), and 5 UPDs (29.41%) (Table 2). In summary, the chromosomal distribution of all the identified CNVs were shown in Figure 2, which is most common in chromosomes 7, 15, 16, X, and 5.

**TABLE 2:** Genomic CNVs of uncertain clinical significance identified by CMA in 21 patients with ID.

Case	Age	Sex	CMA results (ISCN (2013)	Imbalance	Size	No. of genes	Candidate ID genes	Database reported cases^*^
#62	28 M	M	arr[hg19]2p24.2-p24.3(13,675,598–16,756,606) x1	deletion	3.1 Mb	8	DDX1, MYCN	NA
#105	4 M	M	arr[hg19]4q22.1(93,517,978–93,692,360)x1	deletion	174 Kb	1	GRID2	NA
#15	8 Y	M	arr[hg19]4q23(99512154–99523840)x1	Deletion	12 Kb	1 1	TSPAN5	NA
			arr[hg19]11p14.3 (24744166–24757969)x1	deletion	14 Kb		LUZP2	NA
#4	7 Y	M	arr[hg19]5q12.1(60,239,442–60,281,962)x1	deletion	43 Kb	2	NA	NA
#93	9 M	F	arr[hg19]5q14.3-q15(88,654,000–92,564,951)×1	deletion	3.9 Mb	7	NA	2
#95	10 M	M	arr[hg19]7p14.1(40,564,769–40,702,503)x1	deletion	138 Kb	1	NA	NA
#87	24 M	M	arr[hg19]7q36.2(153,524,141–153,744,026)×4	amplification	220 Kb	1	DPP6	1
#30	15 Y	M	arr[hg19]8p23.3(158,048–939,483)×1	deletion	781 Kb	6	DLGAP2	1
#80	13 M	M	arr[hg19]12q24.21-q24.22(116,284,566–117,237,131)x1	deletion	953 Kb	7	MED13L	15
#73	36 M	M	arr[hg19]16p13.3(6,807,673–6,865,110)×1	deletion	57 Kb	1	RBFOX1	1
#34	24 M	F	arr[hg19]16p13.3(6,958,658–7,122,388)×1	deletion	164 Kb	1	RBFOX1	1
#71	6 Y	F	arr[hg19]16p13.3(2,011,148–2,124,718)×3	duplication	114 Kb	10	TSC2, SYNGR3	NA
#1	30 M	M	arr[hg19]18q21.2(52,982,062–53,329,245)x1	deletion	347 Kb	1	TCF4	1
#83	5 Y	M	arr[hg19]20p13(2207150–2368527)x3	duplication	161 Kb	2	TGM6	NA
#74	6 Y	F	arr[hg19]Xp11.22(50,254,924–53,397,510)x1	deletion	3.14 Mb	29	KDM5C, SHROOM4, IQSEC2	3
#42	11 Y	F	arr[hg19]Xp21.1(31,799,595–31,863,313)x1	deletion	64 Kb	1	DMD	1

Age: M, months, Y, years; Sex: M, male, F, female, * indicate the CNVs included in the reported regions here that were reported to be pathogenic or likely pathogenic in the Decipher database.

In order to better analyze the clinical significance of the VUS, the genomic regions were analyzed according to the online tools and database. Nine VUS were found to be larger than the pathogenic or likely pathogenic cases reported in DECIPHER. All the VUS contained at least one protein-coding gene. The tissue expression position, cellular localization, biological process, and mice model were analyzed comprehensively for each gene. Then 18 genes were proposed to support the pathogenicity of these CNVs. As the genes did not fully explain the phenotypic abnormalities in our patients, more experimental evidence was needed *in vitro* or *in vivo*.

As shown in Supplementary Table S1, the clinical features of ID cases with pathogenic/likely pathogenic CNVs or VUS were summarized. Overall, the mean age of the patients was 3.5 years old. Except severe intellectual disability, at least one symptom of neurodevelopmental disorders was detected in the patients. Speech delay and psychiatric disturbances were most common (83.67 and 65.31%, respectively), then seizures (36.73%) and autism spectrum disorder (24.49%). Congenital dysmorphisms (48.98%) and motor developmental delay (30.61%) were the most common symptoms. The segment size of the pathogenic CNVs was common at 1–10 Mb (61.4%).

### Mutations Identified by NGS

**FIGURE 3:**
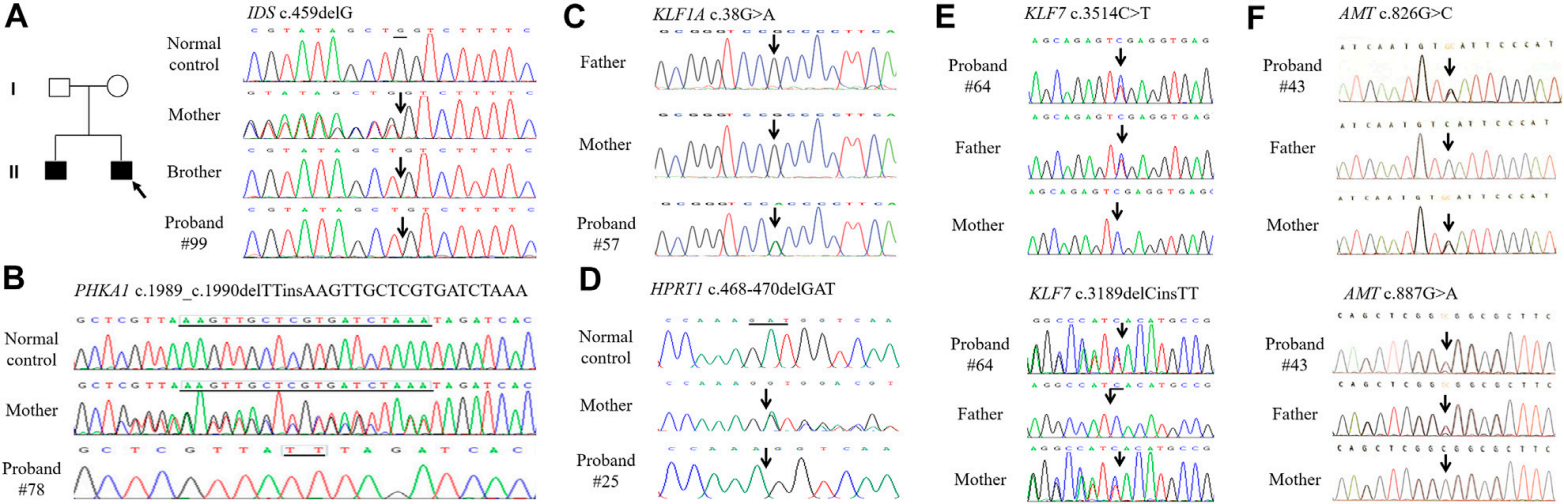
The DNA sequence chromatograms of DNA sequences showing sequence variants in ID patients. **(A)**. Patient #99 and his brother carry heterozygous deletion c.459delG in IDS. **(B)**. Patient #78 carries heterozygous indel c.1989_c.1990delTTinsAAGTTGCTCGTGATCTAAA inherited from the healthy mother in PHKA1. **(C)**. Patient #57 carries *de novo* missense c.38G > A in KIF1A. **(D)**. Patient #25 carries hemizygous deletion c.468_470delGAT in HPRT1. **(E)**. Patient #64 carries compound heterozygous sequence variants c.3514C > T and c.3189delCinsTT in KIF7. **(F)**. Patient #43 carries compound heterozygous sequence variants c.826G > C and c.887G > A in AMT.

The 35 patients with benign CNVs were next tested by WES. According to the international guidelines of ACMG (Richards et al., 2015), 11 SNVs were classified as pathogenic/likely pathogenic and 5 SNVs as VUS in 12 ID cases (Table 3). In which, 12 SNVs were inherited from the parents and 2 were *de novo*. All kinds of SNVs were present, including nine missense variations, four deletions, two nonsense, and one splicing. There were eight compound heterozygous variations, three heterozygous and five hemizygous. Dominant or recessive inheritance could be found, with the recessive in the majority. All these mutations were not found in the normal controls. In total, 9 novel mutations and seven reported mutations were identified in 12 ID cases by using WES (Figure 3).

**TABLE 3:** SNVs identified by WES in 12 patients with ID.

Case	Age	Sex	Phenotype	Gene	Nucleotide change	Protein change	Het	Inheritance	PE	Reported
#43	Neo	M	DR, hypotonia	AMT	c.826G > C	p.Asp276His	C-Het	Pat	P	reported Kure et al. (1998)
					c.887G > A	p.Arg296His		Mat	P	reported Toone et al. (2003)
#99	5 Y	M	GDD, TFF	IDS	c.459delG	p.Trp153fsCysfs*59	Hem	Mat	P	reported Lissens et al. (1997)
#78	32 M	M	GDD, GR	PHKA1	c.1989_c.1990delTTins19NT	p.Tyr663Xfs*1	Hem	Mat	P	this study
#57	4 Y	M	GDD, hyperreflexia	KIF1A	c.38G > A	p.Arg13His	Het	DN	P	reported Tomaselli et al. (2017)
#64	5 Y	M	GDD, spasticity	KIF7	c.3514C > T	p.Arg1172X	C-Het	Pat	LP	this study
					c.3189delCinsTT	p.Ile1063Ilefs*43		Mat	P	this study
#75	6 M	M	GDD, TFF, CHD	CDK13	c.2149G > A	p.Gly717Arg	Het	DN	LP	reported Sifrim et al. (2016)
#25	20 M	M	GDD, dystonia	HPRT1	c.468_470delGAT	p.156_157delLysMetinsLys	Hem	Mat	P	this study
#69	Neo	M	GDD, microcephaly	MCPH1	c.2221CT	p.Arg741X	C-Het	Pat	LP	this study
					c.1759A > G	p.Ile587Val		Mat	VUS	reported
#12	4 M	M	GDD, TFF, dystonia	KDM5C	c.4121 TG	p.Leu1374Arg	Hem	Mat	VUS	this study
#17	3 M	F	GDD, dyskinesia	PLA2G6	c.1427+1G > A	Splicing p	C-Het	Un	LP	reported Wu et al. (2009)
					c.1661C > A	Pro554His		Un	VUS	this study
#19	11 Y	M	ID, ankylosis	IDS	c.484T > C	p.Ser162Pro	Hem	Mat	VUS	this study
#86	5 Y	M	ID, ataxia	CLCN2	c.625G > A	p.Val209Met	Het	Pat	VUS	this study

Age: neo, neonate, M, months, Y, years. Sex: M, male, F, female. Phenotype: GDD, Globe development delay; ID, Intellectual disability; TFF, Typical facial feature; GR, Growth retardation; DR, Decreased responsiveness; CHD, Congenital heart disease; Het, Heterozygosity/Heterozygous C-Het, Compoundheterozygous; Hem, Hemizygous. Inheritance: DN, *de novo*; Mat, Maternal inherited; Pat, Paternal inherited; Un, Untested. Pathogenicity evaluation (PE); P, Pathogenic; LP, Likely pathogenic; VUS, Uncertain clinical significance.

The mean age of these WES-positive patients was 2.85 years old. The most common clinical phenotype was globe development delay (GDD), along with some other manifestations, such as facial dysmorphism, dystonia, and dyskinesia. Most of the cases are known inherited metabolic syndromes, such as Hunter syndrome, Glycogen storage disease, Glycine encephalopathy, and Lesch-Nyhan syndrome. However, for the VUS identified in five cases (#69, #12, #17, #19, and #86), the actual pathogenic mechanism remains to be confirmed by further experiments *in vitro* or *in vivo*.

## Discussion

ID is one of the most common non-structural birth defects in this ethnically diverse region, where economic and social development is particularly unbalanced. According to the incidence rate, there are over 1 million ID patients in Chongqing, most of which without a clear genetic diagnosis. But traditional karyotype analysis, metabolic analysis, and candidate gene screening can only explain a small amount of the causes of idiopathic IDs (Ropers, 2008). With the incidence of chromosomal disease significantly reduced, the proportion of patients with submicroscopic rearrangements increased accordingly (Sagoo et al., 2009; Saldarriaga et al., 2015). Therefore, it is valuable to use high-throughput methods to analyze the genetic etiology of the ID probands (Wright et al., 2018).

Based on a diagnostic evaluation of the phenotype, 1665 ID patients were included. Sixty-one cases were from the department of pediatrics at Xinqiao Hospital and 1,604 cases from the disabled children evaluation center. After phenotypic and medical history analysis, 1,002 cases (62.47%, 1,002/1,604) were found to have known etiological risk factors. Initially, 216 cases were clearly diagnosed by routine genetic analysis. Even with its high diagnostic yield and clinical impact on pediatric care, CMA testing is not yet widely used for clinical diagnostic purposes in children with DD/ID in some districts, including Chongqing. With limited research funds, we strictly enrolled only 105 patients for high-throughput testing. After multi-step screening in 105 undiagnosed ID cases, 56 (53.33%, 56/105) patients were genetically diagnosed by high-throughput techniques. In total, 272 ID cases received a genetic diagnosis, including 196 (72.06%) with chromosomal aberrations, which was the largest proportion. Forty-nine patients (49/272, 18.01%) were identified to carry pathogenic CNVs, and 27 (9.93%) patients were affected by monogenic disorders. The results corresponded to the generally held diagnostic yield of CNVs and SNVs (Stobbe et al., 2014; Bass and Skuse, 2018). The unusually high proportion of chromosomal disorders maybe due to selective bias. These patients were easily recognized and enrolled with a clear molecular diagnosis by conventional karyotyping. For the idiopathic ID cases, clinical application of high-throughput techniques greatly improved the diagnostic yields, which might be a general strategy for the molecular screening of IDs. To date, this is the first report about the etiological distribution of the genetic defects of IDs in Chongqing. The results improve the subsequent genetic counseling and are meaningful to formulate the birth defect prevention and control strategies.

Considering the incidence of syndromes, Trisomy 21 was the most common type, followed by Turner syndrome, Fragile X syndrome, and chromosomal microduplications and microdeletions. The results corresponded to the most common genetic causes of ID. The disabled children evaluation center regularly recruited Down syndrome children for medical guidance and genetic counseling. That might lead to an unusually high diagnosis rate of Down syndrome. In fact, the incidence of Down syndrome has declined significantly in recent years with the introduction of prenatal screening and diagnosis techniques. Typical facial features and globe developmental delay were highly suggestive of the diagnosis of FXS. If there were such specific clues to direct diagnosis, MLPA was the best choice to make a diagnosis. Conventional karyotyping has been widely used to identify the causes of ID patients, because of the advantage in the detection of balanced rearrangements and mosaicism, convenience, and cost-effectiveness. So, MLPA and karyotyping should be optional for ID patients with characteristic facial deformities in the genetic screening department (Miller et al., 2010). In recent years, quantitative fluorescent polymerase chain reaction (QF-PCR) has been widely used in the diagnosis of chromosomal aneuploidy due to its accuracy and high efficiency. The genetic screening results in our cohort also confirmed that QF-PCR/MLPA was an alternative solution. However, the complex rearrangements might also be missed. On this occasion, CMA is sensitive enough to identify such pathogenic CNVs. Therefore, CMA should be recommended as a first-tier clinical diagnostic test for ID patients. WES still has incomparable advantages and cost performance in the identification of SNVs. Briefly, for the appropriate selection of a genetic diagnostic method for ID patients, detailed clinical data and precise clinical diagnosis are extremely important. According to the study, for the syndromic ID patients, a multi-step genetic diagnostic procedure is economical and powerful to identify the genetic defects.

The application of high-throughput techniques has introduced a major advance in the genetic diagnosis of idiopathic ID and associated congenital abnormalities (Mefford et al., 2012; Wiszniewski et al., 2018). However, it is challenging in terms of data interpretation and pathogenicity evaluation. The analysis of data is a time-consuming and labor-intensive work. ACMG updated the technical standards for the interpretation of SNVs and CNVs in 2015 and 2020 separately (Richards et al., 2015; Riggs et al., 2020). Compared to the 2011 version (Kearney et al., 2011), the point-based scoring metric for CNVs paid more attention to the genomic content, the inheritance pattern, and the correlations of clinical findings. With extensive application of NGS-based techniques in clinical laboratories, more abundant variation databases across different races would lead to more consistency across interpretations (Smajlagić et al., 2021; Yuan et al., 2021).

In this study, it was easy to obtain a diagnosis for the recurrent pathogenic CNVs. These regions occur at genomic rearrangement hotspots, chromosomes 7, 15, 16, X, and 5. This is consistent with the results in the Chinese cohorts of pediatric patients with developmental conditions (Yuan et al., 2021). For the likely pathogenic CNVs identified in 14 patients, the pathogenicity evaluation was based on existing reported cases and their absence in the normal population. The parental studies confirmed that most LP CNVs were essentially *de novo*, except case #24. Despite the controversy, case #24 could also be explained by incomplete penetrance. For all the 74 CNVs, more deletions were identified compared to duplications. This might be associated with a bias due to the small sample size. Several patients had large structural abnormalities, which were directedly tested by CMA without prior chromosome analysis. Obviously, the female protective effect for ID patients was also observed in this report. Our study also revealed a group of unique non-recurrent CNVs across the human genome, many of which still warrant further analysis.

For a better clinical interpretation of the VUS, the genomic regions were also analyzed according to the bioinformatic tools and database. Eighteen genes were proposed to support the pathogenicity of these CNVs. According to the literature and databases, some genes have been linked to known neurological syndromes, such as MYCN, MED13L, TCF4, etc. While some other genes were reported to be candidate genes for neurodevelopmental disorders, such as DLGAP2 and RBFOX1 (Chien et al., 2013; Zhao, 2013). In fact, accurate interpretation of the variations still depended on the evidence from functional studies of the candidate genes, by using iPSCs, genetically modified cell lines or mouse models (Zhao and Bhattacharyya, 2018; Fell and Nagy, 2021).

It is estimated that rare SNVs account for approximately 10–20% of ID cases (Vissers et al., 2016; Harripaul et al., 2017). Here in 35 CNV-negative ID patients, 11 pathogenic/likely pathogenic SNVs were identified in 7 cases by WES. Most of the SNVs were inherited from the parents and correlated with GDD phenotype. While for the six VUS identified in five cases, more evidence was needed to determine its pathogenicity. Interestingly, several rare damaging SNVs were also identified in known syndromic ID genes, such as RNASEH2B and EIF2B3. The inheritance pattern of the phenotypes was autosomal recessive, so ACMG interpretation was not suitable for the assessment of these single allelic SNVs. A *de novo* splicing variant in PIP5K1B was also identified, which was reported to be associated with autism (Marshall et al., 2008). However, the actual pathogenic mechanism remains to be further elucidated *in vitro* or *in vivo*. Consistent with previous CMA results, no exon level CNVs were called in the 35 WES cases.

Based on the literature, the underlying genetics of ID/DD seems to be extraordinary complex. A large proportion of patients lacks a specific diagnosis. After using a multi-step screening strategy, nearly half of the 105 patients were still etiologically unknown. According to the technical limitations, the variation located in the non-coding region or the low ratio somatic mosaicism could be missed in the study. The problem might partly be solved by comprehensively introducing whole genome sequencing (WGS), third-generation sequencing (TGS), or an iterative patient-specific approach in the clinic (Lindstrand et al., 2019; Cope et al., 2020). WGS robustly not only captures SNVs and CNVs, but also detects structural variations, STRs (short tandem repeats), ROH (runs of homozygosity), and genomic rearrangements. In certain conditions, WGS could be used as a single test instead of performing CMA followed by WES. In addition, the polygenic genetic basis of the ID or the imperceptible environmental factors might also explain the loss of the heritability.

Certain limitations of the study should be mentioned. The size and composition of the cohort was perhaps the most important one. Since our study was based on previous genetic screening, 1604 ID patients were recruited from the disabled children evaluation center, including 1,002 patients with known etiological risk factors and only 602 patients with genetic screening results. Then 44 patients with negative screening results and 61 idiopathic patients from the pediatric rehabilitation center were strictly selected and tested by CMA and WES sequentially. The 105 cases came from a variety of sources and the sample size is very limited, so some of the findings may be biased. Moreover, most of our results were not further validated by other techniques. We also did not demonstrate a direct relationship between these variations and ID phenotype through functional studies. Therefore, a larger sample size, rigorous inclusion criteria, well-defined multistage screening protocol, and more comprehensive genetic diagnostic approaches are required to obtain a comprehensive overview of the genetic etiology of ID in Chongqing.

In summary, our study explored the genetic etiology of a large ID cohort by using an efficient sequential high-throughput diagnostic strategy. For the strictly selected idiopathic ID cases, CMA and WES might be effective diagnostic tools to greatly improve the diagnostic yields in clinic. These data further extended the variation spectrum of ID in this district, which provided clues for the exploration of neurodevelopmental genes.

## Data Availability

The raw data supporting the conclusion of this article will be made available by the authors, without undue reservation.
