# Supplementary material for: A Tiered Genetic Screening Strategy for the Molecular Diagnosis of Intellectual Disability in Chinese Patients
Source: Front Genet. 2021 Sep 23;12:669217. doi: 10.3389/fgene.2021.669217 (PMC8495063; doi:10.3389/fgene.2021.669217)
Supplement: Supplementary file 1 [file Table1.DOCX]

**Table S1. Summary of clinical features of pathogenic/likely pathogenic CNVs and VUS among our study**

|  | pathogenic/likely pathogenic CNVs | VUS |
| --- | --- | --- |
| Number of Patients | 49 | 21 |
| Mean age ±SD | 2.99±2.31 | 4.75±4.18 |
| Speech delay | 41/49(83.67%) | 13/21(61.9%) |
| Seizures | 18/49(36.73%) | 5/21(23.81%) |
| Psychiatric disturbances | 32/49(65.31%) | 6/21(28.57%) |
| Autism spectrum disorder | 12/49(24.49%) | 4/21(19.05%) |
| Motor developmental delay | 15/49(30.61%) | 7/21(33.33%) |
| Congenital dysmorphisms | 24/49(48.98%) | 8/21(38.1%) |
| CNV segment size | 57 | 17^*^ |
| <100kb | 3/57(5.26%) | 5/17(29.41%) |
| 100kb-1Mb | 8/57(14.03%) | 9/17(52.94%) |
| 1MB-10MB | 35/57(61.4%) | 3/17(17.65%) |
| >10Mb | 11/57(19.3%) | 0/17(0%) |

17^*^: Only the deletion and duplication, not including UPDs.
